# Supplementary material for: Full Soft Capacitive Omnidirectional Tactile Sensor Based on Micro-Spines Electrode and Hemispheric Dielectric Structure
Source: Biosensors (Basel). 2022 Jul 10;12(7):506. doi: 10.3390/bios12070506 (PMC9313071; doi:10.3390/bios12070506)
Supplement: Supplementary file 1 [file biosensors-12-00506-s001.zip › biosensors-1780382-supplementary.pdf]

Supplementary information

1. Figure

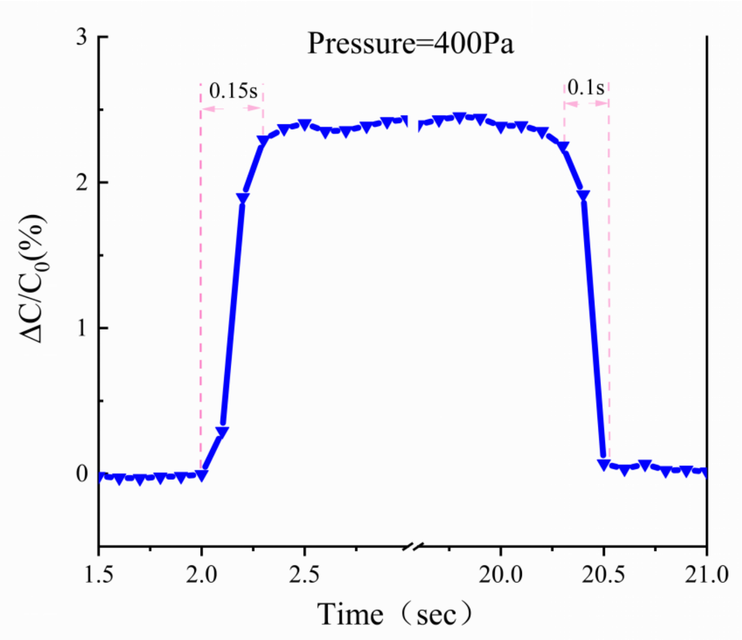

Figure S1. Response time of the sensor.

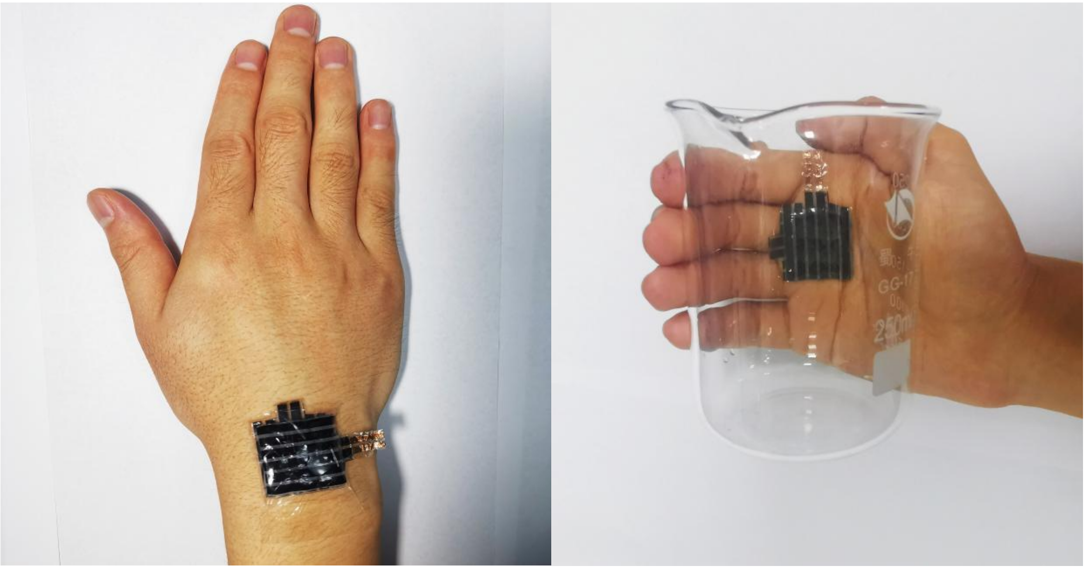

Figure S2. (a). Example of the positioning and fixation of the sensor (fix on wrist joint). (b) Positioning and fixation of the sensor during the breaker experiment.

2. Tables

Table S1. Material parameters of PDMS.

Parameter	Description	Value
E	Young's modulus	850 kPa
ν	Poisson's ratio	0.49
ε_r	Relative permittivity	2.75
t	Thickness of the PDMS film	2 mm

Table S2. Material parameters of Ecoflex.

Parameter	Description	Value
$\boldsymbol{E_1}$	Young's modulus	600 kPa
ν_1	Poisson's ratio	0.49
ε_{r1}	Relative permittivity	2.3
r	Radium of the hemisphere	3.5 mm

Table S3. Performance parameters of LCR tester.

Parameter	Description
Model	TH2829C
Range	0.1 fF~ 9.99 F
Frequency	20 Hz~ 1 MHz
Data point	20 per sec

Table S4. Performance parameters of multi-meter.

Parameter	Description
Model	DM3058E
Range	~ 200 MΩ
Frequency	20 Hz~ 1 MHz
Resolution	$5^{1/2}$ bit
